# ﻿*Plectranthiasraki* (Teleostei, Serranidae), a new species of perchlet from mesophotic coral ecosystems of the Maldives

**DOI:** 10.3897/zookeys.1223.135292

**Published:** 2025-01-16

**Authors:** Bart Shepherd, Hudson T. Pinheiro, Ahmed Najeeb, Claudia R. Rocha, Luiz A. Rocha

**Affiliations:** 1 Steinhart Aquarium, California Academy of Sciences, San Francisco, CA 94118, USA; 2 Department of Ichthyology, California Academy of Sciences, San Francisco, CA 94118, USA; 3 Center for Marine Biology, University of São Paulo, São Sebastião, SP 11600, Brazil; 4 Maldives Marine Research Institute, Ministry of Fisheries and Ocean Resources, Malé 20025, Maldives; 5 Department of Microbiology, California Academy of Sciences, San Francisco, CA 94118, USA

**Keywords:** COI gene, deep reefs, ichthyology, Indian Ocean, rebreather diving, taxonomy

## Abstract

Herein, we describe a new species of *Plectranthias* perchlet found at depths of 100–125 meters in mesophotic coral ecosystems of the Maldives in the Indian Ocean. *Plectranthiasraki***sp. nov.** is unique in both morphology and coloration. The following combination of characters distinguishes it from all known congeners: dorsal fin X, 15; anal-fin rays III, 7; pectoral-fin rays 13 | 13 (13 | 12), all unbranched; principal caudal-fin rays 9 + 8; lateral line complete with 30–32 tubed scales; gill rakers 5 + 12; circumpeduncular scales 11–12; and absence of antrorse or retrorse spines on ventral margin of preopercle. Coloration in life consists of a white to light pink body with two indistinct rows of irregularly shaped red-orange to yellow-orange patches along the dorsal two-thirds of the body, a golden-yellow opercle and maxilla, an indistinct yellow stripe on the dorsal fin, two yellow spots near the base of the anal fin, and two irregularly shaped yellow-orange spots located on either side of centermost caudal-fin rays. With this publication, the genus *Plectranthias* now comprises 67 valid species. This discovery adds to a strong body of research highlighting the novel biodiversity of mesophotic ecosystems, especially in locations like the Indian Ocean, where few prior ichthyological surveys have been conducted.

## ﻿Introduction

The anthiadine genus *Plectranthias* Bleeker, 1873, comprises 66 valid species found in tropical and temperate waters in the Atlantic, Pacific, and Indian oceans (Fricke et al. 2024). In general, they are small (20 cm maximum length, but most in the 5–10 cm range), benthic, feed on small mobile invertebrates, and hide in crevices and holes in relatively deep habitats (depths of 90–420 m) with complex rocky formations (Kuiter 2004; Allen and Walsh 2015). Due to their small size and cryptic habits, they are poorly represented in museum collections, and many species have been described based on a small number of specimens or single individuals (Randall 1980; Heemstra and Randall 2008; Wu et al. 2011; Bineesh et al. 2014; Allen and Walsh 2015; Gill et al. 2016; Shepherd et al. 2018; Gill and Roberts 2020; Shepherd et al. 2020; Wada et al. 2020; Fricke 2021; Koeda et al. 2022). The genus *Plectranthias* is distinguished from other genera in the subfamily Anthiadinae by the following characters: a dorsal fin with 10–12 spines and 13–20 rays, incised between the spinous and soft portions of the dorsal fin; anal fin III, 6–8; pelvic fin I, 5; pectoral fin with 12–18 rays; an absence of auxiliary scales on the head or body; teeth on the palatine and a V- or U-shaped vomer tooth patch, but no teeth on the tongue; 26 vertebrae (rarely 27); and 12–31 total gill rakers (Gill et al. 2021).

Mesophotic coral ecosystems (MCEs), coral reef habitats found at depths of 30–150 m commonly known as the coral reef “twilight zone,” are home to a diversity of organisms that are largely distinct from their shallow-water counterparts (Rocha et al. 2018). While conducting ichthyological and ecological surveys of MCEs at various locations around the globe, our team has encountered many undescribed species, especially from the families Labridae, Pomacentridae and Serranidae (Arango et al. 2019; Shepherd et al. 2019; Tea et al. 2019; Pinheiro et al. 2019; Shepherd et al. 2021). Recent research expeditions in the Maldives have revealed a similar pattern of discovery. In this paper, we describe a new species of *Plectranthias* perchlet seen in MCEs at two atolls in the Maldives.

## ﻿Material and methods

All specimens were collected with hand nets while diving on a mixed-gas closed-circuit rebreather (Hollis Prism 2). Specimens were collected and immediately transported to a field laboratory, where they were photographed, tissues sampled, fixed in 10% formalin, and preserved in 75% ethanol. The preserved specimens were later measured and x-radiographed at the California Academy of Sciences. Measurements were taken with digital calipers to the nearest 0.01 mm and rounded to one decimal place, following the conventions described in Anderson and Heemstra (2012), Williams et al. (2013), and Gill and Roberts (2020). Diagrams in Gill et al. (2021) were especially useful to identify obscure characters. Principal caudal rays are those associated with hypurals. The lowermost principal caudal-fin ray is the ray articulating between the distal tips of the parahypural and the haemal spine of preural centrum 2 as described in Gill et al. (2021). Procurrent caudal-fin rays are those dorsal and ventral to the principal rays. Principal and procurrent caudal-fin ray counts are presented as upper + lower. Vertebral counts are presented as precaudal + caudal. The anterior-most vertebra with a haemal spine was counted as the first caudal vertebra, the urostylar complex the last. Gill raker counts are presented as upper (epibranchial) + lower (ceratobranchial) rakers on the anterior face of the first arch; the angle raker is included in the second count. The anterior supraneural-dorsal ray-pterygiophore-neural spine interdigitation pattern follows Ahlstrom et al. (1976), and is presented as a formula with “0” representing a supraneural, “/” a neural spine, and numerals indicating the number of spines borne by each pterygiophore. The elongated portions of dorsal-fin ray filaments were not included in the measurements, as they were damaged by preservation. Morphometric data for the holotype and paratype is presented in Table 1. Measurements in the text are proportions of standard length (SL) unless otherwise noted. Values in parentheses represent data from the paratype when different from the holotype. The holotype was deposited at the California Academy of Sciences ichthyological collection (**CAS**), and the paratype was deposited at the Natural History Museum of Los Angeles County (**LACM**).

**Table 1.** Morphometric data for *Plectranthiasraki* sp. nov., expressed as a percentage of standard length.

	*Plectranthiasraki* sp. nov.	
	HOLOTYPE	PARATYPE
	CAS-ICH 248439	LACM 61827
Standard length (mm)	66.15	70.41
Head length	44.9	47.7
Greatest body depth	37.4	36.9
Body width	17.1	16.7
Snout length	13.1	12.3
Postorbital of head	25.0	24.6
Bony interorbital width	6.6	6.2
Orbit diameter	9.8	10.0
Upper jaw length	20.0	19.0
Maxilla width	7.5	6.2
Caudal peduncle length	11.7	9.3
Caudal peduncle depth	12.2	11.5
Predorsal length	40.3	38.9
Preanal length	72.9	74.8
Prepelvic length	37.5	39.5
Dorsal fin base length	31.0	47.6
First dorsal spine	5.8	6.3
Longest dorsal spine (number)	16.8 (4^th^ and 5^th^)	18.2 (4^th^)
First segmented dorsal ray	14.7	damaged
Longest segmented dorsal ray- without filament (number)	19.0 (3^rd^)	20.6 (3^rd^)
Anal fin base length	16.8	17.6
First anal spine	8.8	8.6
Second anal spine	19.0	17.4
Third anal spine	15.2	12.8
First segmented anal ray	19.2	18.8
Longest anal spine (number)	11.5 (2^nd^)	10.8 (2^nd^)
Longest segmented anal ray (number)	10.5 (3^rd^)	9.9 (3^rd^)
Caudal fin length	27.0	24.2 (damaged)
Pectoral fin length	34.8	37.5
Pelvic spine length	15.8	15.3
Pelvic fin length	24.8	24.1

Mitochondrial cytochrome *c* oxidase subunit I (COI) DNA was sequenced and analyzed for the new species. DNA extraction and PCR amplification of the COI gene were performed following protocols detailed in Weigt et al. (2012). DNA sequences were compared to the 19 *Plectranthias* species available in GenBank (*P.ahiahiata* Shepherd, Phelps, Pinheiro, Perez-Matus & Rocha, 2018: MH025944; *P.alleni* Randall, 1980: FOAO1479; *P.bennetti* Allen & Walsh, 2015: KT601636; *P.bilaticlavia* Paulin & Roberts, 1987: MN915262; *P.flammeus* Williams, Delrieu-Trottin & Planes, 2013: KC565477–KC565480; *P.fourmanoiri* Randall, 1980: KC567662, KC567663; *P.inermis* Randall, 1980: OQ386015; *P.japonicus* Steindachner, 1883: JQ681323, JQ681324; *P.kamii* Randall, 1980: KU943548; *P.kelloggi* Jordan & Evermann, 1903: KP267643; *P.longimanus* Weber, 1913: JF494178; *P.maculicauda* Regan, 1914: FNZ095; *P.nanus* Randall, 1980: JQ432001–JQ432004, KC565481, KC567661; *P.polygoniu*s Shepherd, Phelps, Pinheiro, Rocha & Rocha, 2020: MN922331; *P.wheeleri* Randall, 1980: LC730852; *P.randalli* Fourmanoir & Rivaton, 1980: KP267613; *P.retrofasciatus* Fourmanoir & Randall, 1979: JN313133; *P.winniensis* Tyler, 1966: KC565482, KC565483; *P.yamakawai* Yoshino, 1972: OP614925) and two in the Barcode of Life Database (*P.ferrugineus* Gill, Pogonoski, Moore & Johnson, 2021: FOAO1382-18; *P.mcgroutheri* Gill, Pogonoski, Moore & Johnson, 2021: FOAO2412-20). Alignments of DNA sequences were done using a standard Geneious global alignment with free end gaps and 65% similarity in the program Geneious Prime 2020.0.3 (Biomatters, Auckland; Kearse et al. 2012).

## ﻿Results

### ﻿Taxonomy

#### ﻿ Plectranthias raki sp. nov.

Taxon classificationAnimaliaPerciformesSerranidae

0EADD278-842A-56CE-8357-56F979292AAE

https://zoobank.org/9B0CEAD6-829B-4BE7-B82C-2AB8E3EBDE77

**Figure 1.**
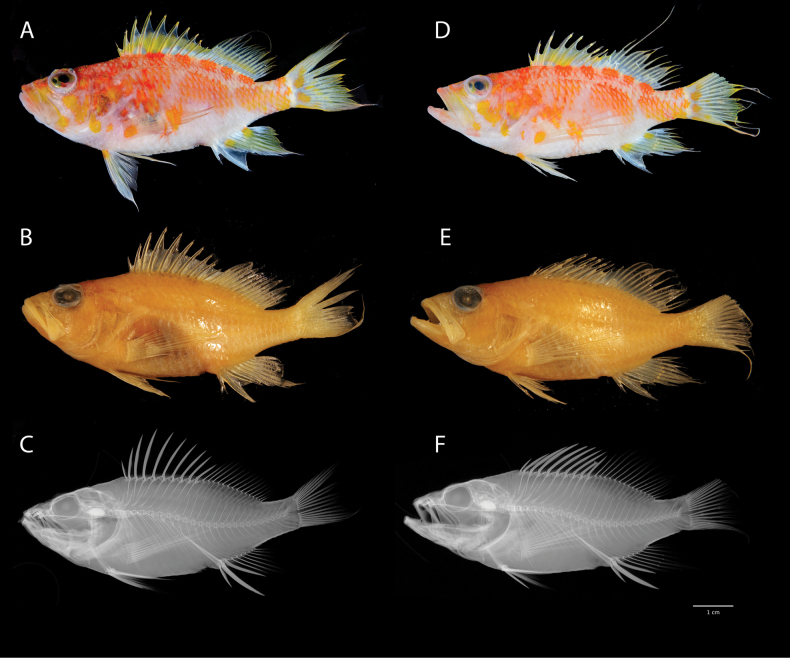
Holotype (left) and paratype (right) of *Plectranthiasraki* sp. nov. Holotype CAS-ICH 248439 66.2 mm SL, shortly after collection (**A**), preserved specimen (**B**) and x-radiograph (**C**). Paratype LACM 61827, 70.4 mm SL, shortly after collection (**D**), preserved specimen (**E**) and x-radiograph (**F**). Photos: **A, D** by Luiz Rocha. **B, C, E, F** by Jon Fong.

**Figure 2.**
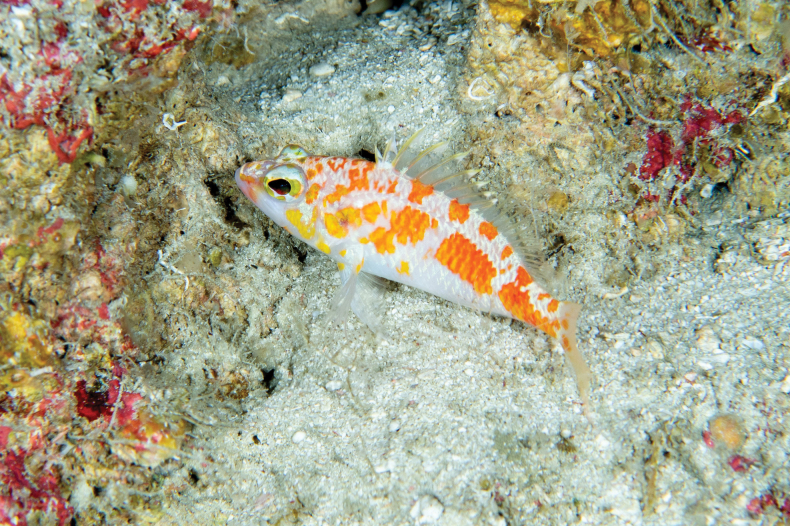
Living specimen (not retained) of *Plectranthiasraki* sp. nov. photographed at 110 m depth at Dhaalu Atoll, Maldives. Photo by Luiz Rocha.

Figs 1 , 2 , Table 1 Dhivehi common name: Raki bureki English common name: Maldivian Perchlet

##### Type locality.

Maldives.

##### Material examined.

***Holotype*.** • CAS-ICH 248439 (Field number LAR2951) 66.2 mm SL, GenBank PQ416576. Location: Kuramathi Outer Reef, Rasdhoo Atoll, Maldives 4°15'22"N, 72°59'00"E, depth of collection 118 m, collected with hand nets by B Shepherd, HT Pinheiro, MV Bell, and LA Rocha, 9 December 2022. ***Paratype*.** • LACM 61827 (Field number LAR2952) 70.4 mm SL, GenBank PQ416577. Same collection data as holotype.

##### Diagnosis.

*Plectranthiasraki* sp. nov. is unique in both morphology and coloration. The following combination of characters distinguishes it from all known congeners: dorsal fin X, 15; anal-fin rays III, 7; pectoral-fin rays 13 | 13 (13 | 12), all unbranched; principal caudal-fin rays 9 + 8; lateral line complete with 30–32 tubed scales; 3 supraneural bones, predorsal formula 0/0 + 0/2/1 + 1/1/1/; gill rakers 5 + 12; circumpeduncular scales 11–12; oblique rows of scales on cheek 8 (7); longest dorsal spine the 4^th^ or 5^th^; no fleshy tips on the dorsal-fin spines; no antrorse serrations on preopercle. Live coloration consisting of a series of irregularly shaped patches of red-orange along dorsal two-thirds of body; patches divided into two indistinct rows by the lateral line; patches red-orange dorsally and posteriorly, becoming more yellow-orange anteriorly and ventrally, golden yellow on opercle and maxilla; anal fin pointed, mostly white proximally, yellow distally, with two yellow spots approximately one-third orbit diameter at base of third spine and fifth and sixth soft rays; two irregularly shaped yellow-orange spots, approximately one-third orbit diameter, located on either side of centermost caudal-fin rays; small orange spot, approximately one-half orbit diameter, at base of pelvic fin.

##### Description.

Dorsal rays X, 15, all segmented rays branched; anal rays III, 7, all segmented rays branched; pectoral-fin rays 13 | 13 (13 | 12), all unbranched; pectoral fin moderately long, longest ray reaching to vertical above midpoint of anal fin; pelvic-fin I, 5; upper procurrent caudal-fin rays 6; lower procurrent caudal-fin rays 4; principal caudal-fin rays 9 + 8; branched caudal-fin rays 9 + 7 (8 + ?); lateral line complete with 32 (30) tubed scales on the left side; scales above lateral line to origin of dorsal fin 3; scales above lateral line to base of middle dorsal spine 2; scales below lateral line to origin of anal fin 10; oblique rows of scales on cheek 8 (7); circumpeduncular scales 11 (12); gill rakers 5 + 12, the upper 4 and lower 3 rudiments; pseudobranchial filaments 18 (15); branchiostegal rays 7. Vertebrae 10 + 16; supraneural (predorsal) bones 3; predorsal formula 0/0 + 0/2/1 + 1/1/1/; dorsal pterygiophores in interneural spaces 9–13 1/1/1+1/1+1/1; no trisegmental pterygiophores associated with dorsal fin; terminal dorsal pterygiophore in interneural space 18; no trisegmental pterygiophores associated with anal fin; terminal anal pterygiophore in interhaemal space 5; ribs present on vertebrae 3 through 10; epineurals present on vertebrae 1 through 12 (possibly 13); parhypural and hypurals autogenous; well-developed hypurapophysis on parhypural; epurals 3; single uroneural (posterior uroneural absent); ventral tip of cleithrum with well-developed posteroventral process; proximal tip of first anal-fin pterygiophore near distal tips of parapophyses on vertebra 10.

Body moderately deep, the depth 2.7 in SL, and compressed, the width 2.2 in depth; head fairly short, 2.2 (2.1) in SL; dorsal fin originates at a vertical line just above the third lateral line scale; dorsal fin continuous and notched between the spinous and soft portions to about half of the length of the first soft ray; dorsal-fin spines without fleshy tabs on the tips; dorsal-fin base length 3.2 (2.1) in SL; the fourth and fifth dorsal spines the longest and the same length in the holotype (the fourth the longest on the paratype); third dorsal-fin soft ray the longest, with extended filament; anal-fin base length 6.0 (5.7) in SL; second anal-fin spine the longest; anal fin pointed with the third segmented ray the longest; pectoral fin moderately long and pointed, 2.9 (2.7) in SL; pelvic fin relatively short, 4.0 (4.2) in SL, and not reaching anus; caudal fin slightly emarginate, with several filaments on elongated ray branches (caudal fin in paratype damaged); caudal peduncle length 3.9 (5.1) in HL; caudal peduncle depth 3.7 (4.2) in HL.

Morphometric values are summarized in Table 1.

Head of moderate size, 2.2 (2.1) in SL; snout pointed, 3.4 (3.9) in HL; mouth relatively large, terminal and oblique, the posterior margin of maxilla reaching to vertical line almost directly beneath the center of pupil; upper jaw length 2.3 (2.5) in HL; maxilla expanded posteriorly; upper jaw with one large canine on each corner; 5 irregular rows of villiform teeth, largest in back, the teeth on the last row as big as the front canines; lower jaw with one small canine on either side of symphysis and 4–5 irregular rows of villiform teeth, the largest in the back; a pair of enlarged canines on each side of the dentary; teeth on the last row as big as the canines; palatine with 4 rows of small villiform teeth; vomer with 5 rows of villiform teeth.

Opercle with 3 spines, the middle one the largest, sharp and pointed, and terminating most posteriorly, the upper one obscured by scales; posterior margin of preopercle with 26 serrae; ventral margin of preopercle smooth and without conspicuous antrorse or retrorse spines; posterior margins of interopercle and subopercle smooth, obscured by scales; posttemporal with 3 small serrations; lower margin of infraorbital 1 smooth; anterior nostril located close to the anterior margin of orbit, with a small flap; posterior nostril located adjacent to anterior border of orbit, without flap.

Scales ctenoid with peripheral cteni; lateral line broadly arched over pectoral fin, following body contour beneath dorsal fin to caudal-fin base; scales between eyes; no scales on maxilla, chin, mandible, lower part of snout, or branchiostegal rays; scales on head starting above the center of the eyes; triangular shaped patch of 6 rows of scales on pectoral fin, extending approximately ¼–⅓ length of fin, extending furthest on 7^th^ and 8^th^ rays; scales on basal fourth of caudal fin; anal fin with one row of scales along anterior half of base; all other fins without scales.

***Coloration when fresh***: (Figs 1A, C, 2) Body pinkish-white with a series of irregularly shaped patches of red-orange along dorsal two-thirds of body; patches red-orange dorsally and posteriorly, becoming more yellow-orange anteriorly and ventrally; patches are golden yellow on opercle, especially on maxilla; one indistinct yellow stripe crossing opercle diagonally from bottom edge of eye; indistinct orange-red stripe extending from anterior margin of eye to upper lip; orbit white with indistinct yellow stripe through pupil, a continuation of the red-orange stripe originating at the snout tip; sharp break in orange-colored body-patches along lateral line, forming two distinct rows; this is especially pronounced in living specimens; patches above lateral line smaller and associated with pairs or trios of dorsal-fin spines and rays, creating an alternating pattern of five orange spots interspersed with white areas along the base of dorsal fin; patches below the lateral line are larger and more irregular in shape and distribution anteriorly, more rectangular and uniform on posterior half of body; throat and belly white; dorsal fin white at base; interspinous membranes of dorsal fin mostly yellow, white proximally, hyaline distally; membranes of soft dorsal mostly white with yellow stripe roughly midway from body and following contour of body; dorsal spines yellow, rays with yellow tips; anal fin mostly white proximally, yellow distally, with two yellow spots approximately one-third orbit diameter at base of third spine and fifth and sixth soft rays; anal-fin spines white; pelvic fins white proximally, hyaline distally, with yellow-orange spot at base of spine; pectoral fin white to hyaline, with orange spot, approximately one-half diameter of orbit, at fin base; caudal fin white at origin, becoming pale yellow distally, with two irregularly shaped yellow-orange spots, approximately one-third orbit diameter, located on either side of centermost fin rays; additional, smaller spots of similar coloration distally; tips of caudal-fin rays yellow; proximal half of dorsalmost and ventralmost caudal-fin rays yellow-orange.

***Color in alcohol***: Uniform pale golden-brown with no distinct markings. Scattered melanophores on the nape and along base of dorsal fin (denser on the nape).

##### Etymology.

The species name, raki, means “feeling shy to confront people” in the Dhivehi language. This was chosen because *Plectranthias* are shy by nature and typically hide from us when we are conducting surveys. To be treated as a noun in apposition.

##### Distribution and habitat.

*Plectranthiasraki* sp. nov. is known only from the Maldives, where it is likely widespread. It was seen inhabiting small holes of reef walls at several locations between Rasdhoo (4°15'N, 72°57'E) and Dhaalu atolls (2°41'N, 72°51'E) at approximately 100 to 125 m depth.

## ﻿Discussion

The Maldives Archipelago shelters a rich biodiversity of reef fishes. However, only *Plectranthiaswinniensis* Tyler, 1966 was previously known for the region (Randall and Anderson 1993). *Plectranthiasraki* is distinguished from *P.winniensis* by the number of pectoral-fin rays (12–13, versus 16–18), tubed lateral line scales (32, versus 8–27), and in coloration, especially in having a yellow stripe on the dorsal fin and lacking a white spot at the caudal peduncle, as in *P.winniensis*. Four species, *Plectranthiasgarrupellus* Robins & Starck, 1961, *Plectranthiashinano* Shepherd, Phelps, Pinheiro, Rocha & Rocha, 2020, *Plectranthiaskojiorum* Koeda, Muto & Wada, 2021, and *Plectranthiaslongimanus* Weber, 1913, share the following diagnostic characters with *Plectranthiasraki*: dorsal fin X, 15; anal fin III, 7; and pectoral-fin rays 12–13. In addition to the morphological differences that follow, *P.raki* is easily distinguished from these four species based on its living coloration. *Plectranthiasraki* can be distinguished from *Plectranthiasgarrupellus* by having 30–32 tubed lateral-line scales, compared to 28–29 in *P.garrupellus*, and by lacking antrorse spines on the preopercle (versus 2 spines in *P.garrupellus*). *Plectranthiasraki* has 5 gill rakers on the upper arch compared to 7–8 in *P.hinano*, a shorter dorsal-fin base length, 31% SL versus 48.5% in *P.hinano*, and lacks antrorse spines on the preopercle, whereas *P.hinano* has 3 antrorse spines. *Plectranthiasraki* has 16 branched caudal-fin rays, while *Plectranthiaskojiorum* has only 13. *Plectranthiasraki* has 3 supraneural bones and lacks antrorse spines on the preopercle, while *P.kojiorum* has 2 predorsal bones and 2 antrorse spines. *Plectranthiasraki* differs from *P.longimanus* by having a complete lateral line with 30–32 tubed scales, compared to the incomplete line of 12–15 tubed scales in *P.longimanus*.

*Plectranthiasraki* is similar in many counts and measurements to *Plectranthiasklausewitzi* Zajonz, 2006 from the Red Sea, but differs in having a longer snout length (13.1% SL versus 10.7% SL in *P.klausewitzi*, a smaller orbit diameter (9.8% SL in *P.raki* versus 13.9% in *P.klausewitzi*), and by having fewer pectoral-fin rays (12–13 in *P.raki* versus 14–15 in *P.klausewitzi*). The living coloration of *Plectranthiasklausewitzi* is unknown at this time.

*Plectranthiasraki* is distinct from the other Indian Ocean species, *Plectranthiasalcocki* Bineesh, Gopalakrishnan & Jena, 2014, *Plectranthiasalleni* Randall, 1980, and *Plectranthiasmorgansi* Smith, 1961 based on the following characteristics. *Plectranthiasraki* has 12–13 pectoral-fin rays and 30–32 tubed lateral-line scales, and three scales above the lateral line to the origin of the dorsal fin, while *P.alcocki* has 14 pectoral-fin rays, 28 tubed lateral-line scales, and only one scale above the lateral line to the origin of the dorsal fin. *Plectranthiasraki* differs from *P.alleni* in dorsal-fin counts (X, 15 versus X, 14), pectoral-fin rays (12–13, versus 15–17), the number of circumpeduncular scales (11–12 versus 14–15), and in coloration, by lacking the short narrow dark stripe in front of the eye and the faint dusky stripe from behind the eye across the upper side of the body that distinguishes *P.alleni*. *Plectranthiasraki* differs from *Plectranthiasmorgansi* by having the 4^th^ dorsal spine the longest (versus the 3^rd^ in *P.morgansi*, which also has a pennant), shorter pelvic fins (not reaching the anus, as in *P.morgansi*), pointed anal fin (versus rounded in *P.morgansi*) and an emarginate caudal fin (versus rounded in *P.morgansi*) (Smith 1961).

Our specimens also resemble an undescribed species of *Plectranthias* collected in the Andaman Sea on the Tanintharyi coast of Myanmar (Gill and Psomadakis 2018). However, there are key differences that distinguish them. *Plectranthiasraki* has 12–13 pectoral-fin rays versus 14 on the Myanmar specimen, and lacks the two enlarged antrorse spines on the lower part of the preopercle seen on the specimen from Myanmar. In addition, there are several morphometric differences, including a smaller orbit diameter (9.8% SL in *Plectranthiasraki* versus 14.9% SL in the specimen from Myanmar), a shorter caudal peduncle (11.7% SL versus 20.4% SL in the Myanmar specimen), shorter pectoral fins (34.8% SL in *Plectranthiasraki* versus 42.9% SL in the Myanmar specimen), and the longest segmented dorsal ray being the 3^rd^ in *Plectranthiasraki* and the 7^th^ in the specimen from Myanmar. A second undescribed species, also collected in Myanmar, is documented in the same publication (Gill and Psomadakis 2018). The specimen was subsequently lost, so there are limited details for comparison, but it is also distinguishable from *Plectranthiasraki* morphologically and in coloration. *Plectranthiasraki* has 12–13 pectoral fin rays, while the second specimen from Myanmar has 14. *Plectranthiasraki* also has a predominately white caudal fin with two indistinct, but pronounced, yellow-orange spots, while the caudal fin on the second specimen from Myanmar is solid yellow, and *Plectranthiasraki* lacks the red marking on the middle spines of the dorsal fin as seen in the photograph of the specimen from Myanmar.

The barcode fragment of the COI gene of *Plectranthiasraki* is not within 10% distance to any COI sequences of *Plectranthias* available on GenBank and the Barcode of Life Database. Therefore, we couldn’t identify a close genetic relative. However, the new species seems to form a group with other distantly related species (12–15% genetic divergence) that include *P.bennetti*, *P.hinano*, *P.ferrugineus*, and *P.kojiorum*. Since DNA sequences are available for just about 30% of the species in the genus, we prefer not to discuss their relationships here in detail because they will likely change with the addition of more species.

## Supplementary Material

XML Treatment for Plectranthias raki

## ﻿Additional information

### Author ORCIDs

Bart Shepherd https://orcid.org/0000-0002-8918-1551

Hudson T. Pinheiro https://orcid.org/0000-0002-3143-1474

Claudia R. Rocha https://orcid.org/0000-0001-8069-6535

Luiz A. Rocha https://orcid.org/0000-0003-4011-569X

### Data availability

All of the data that support the findings of this study are available in the main text.
